# Computer Vision-Based Gait Recognition on the Edge: A Survey on Feature Representations, Models, and Architectures

**DOI:** 10.3390/jimaging10120326

**Published:** 2024-12-18

**Authors:** Edwin Salcedo

**Affiliations:** Department of Mechatronics Engineering, Universidad Católica Boliviana “San Pablo”, La Paz 4807, Bolivia; esalcedo@ucb.edu.bo

**Keywords:** gait recognition, gait analysis, edge computing, computer vision, edge AI

## Abstract

Computer vision-based gait recognition (CVGR) is a technology that has gained considerable attention in recent years due to its non-invasive, unobtrusive, and difficult-to-conceal nature. Beyond its applications in biometrics, CVGR holds significant potential for healthcare and human–computer interaction. Current CVGR systems often transmit collected data to a cloud server for machine learning-based gait pattern recognition. While effective, this cloud-centric approach can result in increased system response times. Alternatively, the emerging paradigm of edge computing, which involves moving computational processes to local devices, offers the potential to reduce latency, enable real-time surveillance, and eliminate reliance on internet connectivity. Furthermore, recent advancements in low-cost, compact microcomputers capable of handling complex inference tasks (e.g., Jetson Nano Orin, Jetson Xavier NX, and Khadas VIM4) have created exciting opportunities for deploying CVGR systems at the edge. This paper reports the state of the art in gait data acquisition modalities, feature representations, models, and architectures for CVGR systems suitable for edge computing. Additionally, this paper addresses the general limitations and highlights new avenues for future research in the promising intersection of CVGR and edge computing.

## 1. Introduction

In an era marked by the proliferation of smart devices and video surveillance systems, biometrics has emerged as a crucial field for secure and convenient identity verification. Among various biometric modalities, computer vision-based gait recognition (CVGR), which analyses an individual’s unique walking pattern, provides a non-invasive and potentially unobtrusive identification method [1]. However, traditional CVGR systems, often reliant on centralised cloud-based architectures, face challenges in terms of latency, bandwidth consumption, and privacy concerns. The emergence of edge computing, which brings computation and data storage closer to the source, presents a compelling paradigm shift. CVGR systems deployed on the edge unlock diverse opportunities, from reducing latency in biometric authentication to enabling real-time fall detection and rehabilitation monitoring and even opening new avenues for human–computer interaction.

**Figure 1:**
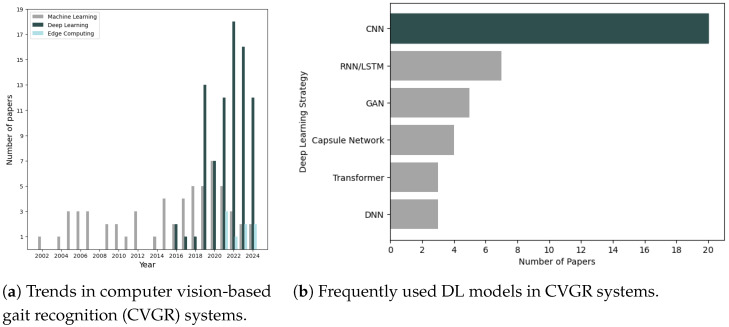
A comparative analysis illustrating the growing preference for DL architectures. The illustrations summarise the findings from the papers reviewed in this survey.

Gait data can be gathered using various wearable sensors, including accelerometers, gyroscopes, and goniometers [2]. In contrast, CVGR systems primarily rely on 2D or 3D imaging sensors to capture gait data from long distances, even without the subject’s active participation [1]. CVGR systems offer several advantages: they are non-invasive, are robust to impersonation, function well over long distances, do not need high-quality cameras, and can be combined with other sensing modalities [3]. Additionally, as shown in Figure 1a, recent years have witnessed a shift in gait pattern recognition, moving away from traditional approaches towards modern techniques based on deep learning (DL). Not only does this enable higher accuracy in gait identification, but it also allows the deployment of CVGR in more challenging and varied environments.

Despite advancements, challenges such as varying view angles, intra-class variations, body segment localization, and occlusions persist in CVGR systems. Researchers have recently addressed some of these challenges by increasing the complexity of the architectures [4,5,6,7]. However, such complexity comes at the expense of high computational costs and resource requirements, hindering on-site deployment due to high resource demands. Fortunately, ongoing advancements in hardware and software for embedded systems are leading to smaller, more powerful computers (e.g., Jetson Nano Orin, Jetson Xavier NX, Khadas VIM4, and Google Coral Edge TPU), enabling the embedding of DL models, even in resource-constrained settings. This trend positions edge computing as a promising solution for deploying DL-based gait recognition in applications like intelligent, real-time surveillance, and healthcare monitoring.

Several comprehensive reviews have been conducted on CVGR systems, focusing on DL [8], appearance-based gait recognition [9], and energy image-based gait recognition [10]. However, to the best of the authors’ knowledge, no comprehensive review has specifically addressed the intersection between gait recognition systems based on computer vision and edge computing. This gap in the literature highlights the value of the present work. Therefore, we conducted a thorough survey of CVGR systems, with a focus on 2D imaging-based gait recognition due to its suitability for low-resource end devices and widespread use in video surveillance systems. The survey examines the benefits, applications, and opportunities associated with deploying CVGR on edge devices. It also explores the obstacles to such deployment, considering factors such as computational efficiency, real-time performance, and data security.

**Figure 2:**
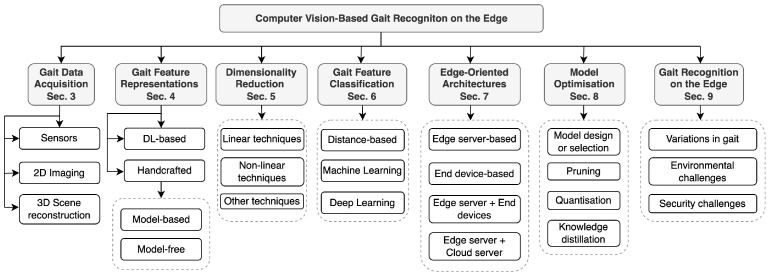
General structure of this survey paper and our proposed taxonomy of the existing technologies that facilitate on-device deployment of CVGR systems for real-time recognition.

The structure of this survey is illustrated in Figure 2. Firstly, Section 2 presents a summary of the review process and inclusion/exclusion criteria. Then, Section 3 details common sensors and methods for acquiring gait data. Following this, Section 4 explores various techniques for representing and processing gait features in CVGR systems. We then delve into different dimensionality reduction techniques aimed at achieving computational efficiency. Section 6 provides a detailed overview of both traditional and modern models for gait feature classification. Next, Section 7 examines various edge computing architectures, while Section 8 presents techniques to accelerate machine learning inference. The convergence of gait recognition and edge computing is discussed in Section 9. Finally, the paper concludes by discussing and summarising our findings in Section 10 and Section 11.

## 2. Survey Methodology

The present section elucidates the methodology used to review recent publications on CVGR systems suitable for edge computing.

### 2.1. Research Questions

The investigation was oriented to the following research questions (RQs):

- RQ1: What are the primary sensors for gait data acquisition utilised in CVGR systems?
- RQ2: What are the known gait feature extraction, reduction, and representation approaches and their associated challenges in CVGR systems?
- RQ3: Which gait feature classification framework outperforms others, and what techniques can be used to mitigate the challenges associated with these frameworks?
- RQ4: What are the architecture options, challenges, and good practices when it comes to deploying CVGR systems on the edge?
- RQ5: What are the current limitations of CVGR systems based on edge computing, and what are the potential areas for future improvement?

### 2.2. Search Strategy and Inclusion/Exclusion Criteria

This review begins by establishing a foundation in both CVGR systems and edge computing-based inference architectures. It then explores the intersection of these fields, identifying key research gaps. To achieve this, we conducted a literature review of papers published between January 2000 and November 2024. While we prioritised recent literature, we also included seminal works to provide a comprehensive theoretical context on traditional gait recognition approaches for Section 4 and Section 5.

The literature review encompassed a manual search of relevant publications in key databases, including SCOPUS, Mendeley, Science Direct, Springer Link, IEEE Xplore, and ACM Digital Libraries. We employed a targeted keyword-based approach for each section of the review. For instance, when identifying articles on data acquisition methods for gait recognition (Section 3), we combined keywords such as “depth sensing” AND “gait recognition”. Similarly, for our examination of gait recognition on the edge (Section 9), we used search strings like “gait recognition” and (“edge computing” or “edge AI”) or “gait recognition” and “deployment”. This strategy was applied consistently throughout the review, along with a temporal filter to limit the search to relevant publications. To ensure the quality and relevance of the literature review, we applied the following filtering criteria:

- Included only articles published in English.
- Prioritised peer-reviewed papers based on relevance to the CVGR and edge computing fields, excluding those with weaker or less pertinent contributions.
- Prioritised papers indexed in Scopus that explored the use of CVGR systems in biometric applications, where low latency is crucial.
- Prioritised papers presenting novel methodologies for CVGR systems on the edge.
- Excluded papers lacking sufficient experimental results or methodological details.
- While focusing primarily on CVGR, we also included studies on gait recognition using other sensor modalities for comparative analysis.
- Selectively included papers published before 2020, focusing on those that introduced original ideas or made significant advancements in gait recognition.

## 3. Data Acquisition Methods for Gait Recognition

Gait recognition systems have traditionally relied on two primary data collection methods: 2D imaging and sensor-based approaches, the latter often employing Internet of Things (IoT) infrastructure or wearable devices. However, researchers are increasingly exploring alternative methods, such as depth sensing and 3D scene reconstruction [8]. This section explores both traditional and emerging gait data acquisition methods.

### 3.1. Sensor-Based Methods

Various sensors have been explored for gait recognition. For instance, floor sensors are commonly employed to register the pressure exerted by walking individuals, converting these data into signals [11,12]. These signals are then processed to either distinguish unique gait characteristics among individuals or identify gait anomalies. However, the high cost of deploying floor sensors often necessitates controlled indoor environments. Similarly, wearable sensors, such as accelerometers, gyroscopes, goniometers, and electromyography, have also been applied to gait analysis [2]. These sensors, connected wirelessly or with wires to embedded devices, smartphones, or other wearables, capture dynamic parameters of the human body for gait analysis [2]. For instance, researchers in [13] recently explored edge computing for gait recognition using gyroscope data. They deployed a four-layer convolutional neural network (CNN) on an Arduino Nano 33 BLE, a smartphone, and a Brainchip Akida processor, achieving real-time person identification. Despite this progress, this review does not discuss works developed with this sensing modality because they require subject cooperation.

### 3.2. 2D Imaging-Based Methods

CVGR systems based on 2D imaging utilise 2D image or video data of human gait sequences, typically captured via a digital camera, and analyse these data using 2D image or video processing techniques. This sensing modality has been widely researched, leading to the creation of several well-established datasets for development and performance evaluation, some of the most representative of which are listed in Table 1. The choice of specific image processing techniques after data acquisition is significantly influenced by the design of the target CVGR system. Common processing methods include the following:

- Noise reduction to minimise the impact of noise or artifacts in the video data.
- Background subtraction and silhouette extraction to isolate the moving human subject (foreground) from the static background.
- Gait cycle detection and normalisation to identify and standardise the repetitive pattern of gait cycles.

The present review primarily focuses on this sensing approach due to the advantages of processing imaging data on resource-constrained devices, including faster computation and the ability to utilise low-cost, standard cameras. Additionally, it is worth noting that video surveillance systems, which are more widely used nowadays, use mainly 2D cameras. Therefore, this enables the easy integration of CVGR systems into current video surveillance systems, embedded systems, and mobile devices.

### 3.3. 3D Scene Reconstruction-Based Methods

While 2D image sensors are the foundation of computer vision systems, other sensors can also play an important role, particularly in 3D scene reconstruction. For instance, technologies such as depth sensing, 3D lasers, and stereo vision aim to capture the complete 3D structure of real-world objects and have recently shown promising results for gait recognition. However, their current high computational demands and associated costs limit their widespread deployment in edge computing-based architectures.

#### 3.3.1. Depth Sensing

CVGR systems have leveraged depth cameras (e.g., Microsoft Kinect, Zed, and Intel RealSense) due to their ability to provide gait information based on a skeleton model [26,27,28,29]. This approach, also known as model-based gait recognition, utilises both RGB images and depth maps to identify joint points in the human body. It then models the anatomical components to derive a unique gait signature [30]. Because they are less sensitive to lighting variations and occlusion issues, depth cameras provide a more consistent input for gait recognition. However, several studies have found this approach to be computationally expensive, particularly due to the need for high-resolution images for accurate joint point identification, which limits its implementation in real-time detection tasks, particularly in uncontrolled scenarios [31]. Despite these limitations, recent progress suggests promising potential for future applications of depth sensing in CVGR systems at the edge.

#### 3.3.2. 3D Lasers

Recent investigations have explored 3D and 4D data acquisition to represent the human body’s surface during walking or other movements. These data can be obtained using various technologies, including 3D lasers, radar signals [5], and light detection and ranging (LiDAR) technologies [32], among others. These approaches aim to obtain point clouds, which are sets of data points in 3D space, each with X, Y, and Z coordinates, that provide fine-grained geometric features of a person’s body. Furthermore, 4D point clouds extend 3D point clouds by incorporating time as the fourth dimension, allowing for the capture of both spatial and temporal aspects of a person’s gait [33]. Consequently, this enables the capture of dynamic walking patterns over a period, facilitating a more detailed analysis of temporal variations in movement.

Among the aforementioned sensors, LiDAR has emerged as a popular choice due to its reliability in capturing gait information even in challenging situations like low light or occlusions. Recent LiDAR-based models have demonstrated compelling results compared to 2D imaging. One example is LidarGait, proposed by Shen et al. [32], which converts sparse point clouds from LiDAR data into depth maps, enabling the capture of the 3D shape of a person’s gait. To address the scarcity of LiDAR gait data, the authors created SUSTech1K, a dataset comprising 25,239 gait sequences from 1050 subjects, covering various factors like visibility, viewing angles, occlusions, clothing, carrying conditions, and scene variations. Challenges for 3D laser-based gait recognition include occlusions, variations in clothing, changes in viewpoint, and the high cost of data acquisition devices, all of which represent important areas for future research [8].

#### 3.3.3. Stereo Vision

Stereo vision enables the reconstruction of 3D scenes through triangulation. Specifically, it uses two or more cameras to capture images of the same scene from slightly different viewpoints, enabling the perception of depth maps with gait information. For example, in the medical field, researchers in [34] have applied stereo vision to automate the analysis of screening tests—namely, the Timed Up and Go (TUG) and Performance Oriented Mobility Assessment (POMA) tests—administered to elderly individuals. Their aim was to fuse 2D gait data with depth information in order to identify abnormalities that may indicate health risks or mental health decline, achieving compelling results in real daycare facilities. For gait recognition as a biometric tool, the authors of [35] proposed a stereo-vision pipeline that extracts 3D contours from a person’s silhouette in a video sequence, calculates a “stereo gait feature” (SGF) from these contours, reduces dimensionality using principal component analysis, and then classifies gait patterns using neural networks.

While powerful for 3D perception, stereo vision is not ideal for CVGR systems. This is mainly because it demands significant computational resources for real-time 3D scene reconstruction, a major drawback for systems with limited processing power, such as those deployed in real-world settings [36]. Furthermore, the accurate calibration of the stereo camera setup is also critical. Without it, depth information becomes unreliable, leading to significant errors in depth estimation and impacting gait recognition accuracy. In contrast, surveillance cameras (RGB and grayscale) are increasingly common due to their low cost and easy installation. They are now prevalent in urban areas, businesses, and public spaces, with growing use in rural areas, private homes, and even natural environments [37]. Therefore, the present article focuses on 2D imaging-based data acquisition.

## 4. Feature Representations for Computer Vision-Based Gait Recognition

**Figure 3:**
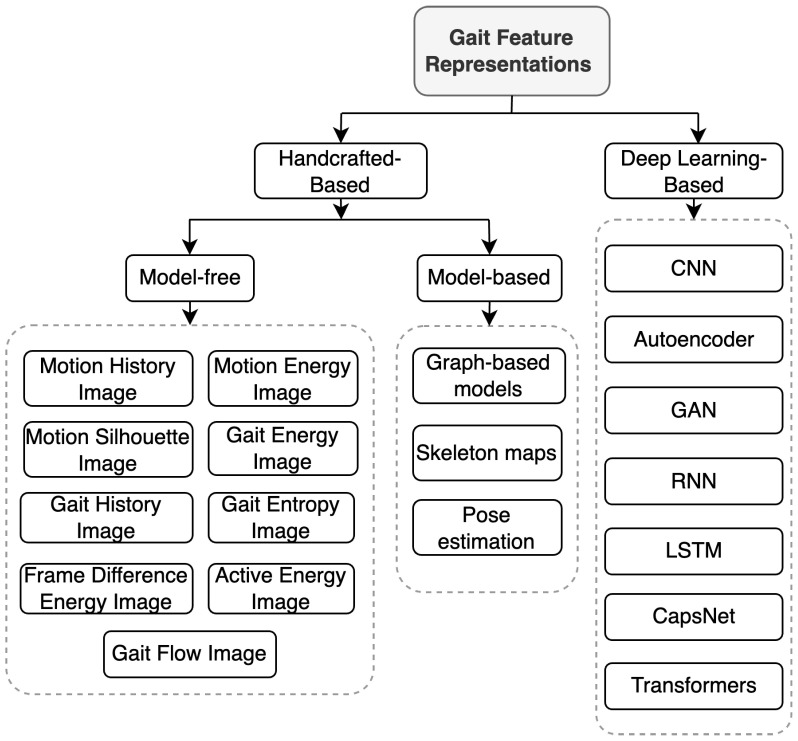
Broad perspective on gait feature representations.

Extracting features from 2D imaging data is crucial in CVGR systems. This step creates feature representations that capture the unique qualities of a person’s walk. As shown in Figure 3, feature extraction methods fall into two main categories: DL-based and handcrafted. DL-based methods automatically extract features from raw gait data (e.g., images, video frames, or silhouettes) using DL models, such as CNNs, recurrent neural networks (RNNs), or long short-term memory networks (LSTMs). These models can be trained end-to-end, integrating feature extraction and gait classification into a single optimised process. However, it is worth noting that DL models are also used to process and classify handcrafted features. Section 6.2 provides a more detailed exploration of DL-based feature extraction and common DL models, including their strengths and weaknesses for gait recognition.

**Figure 4:**
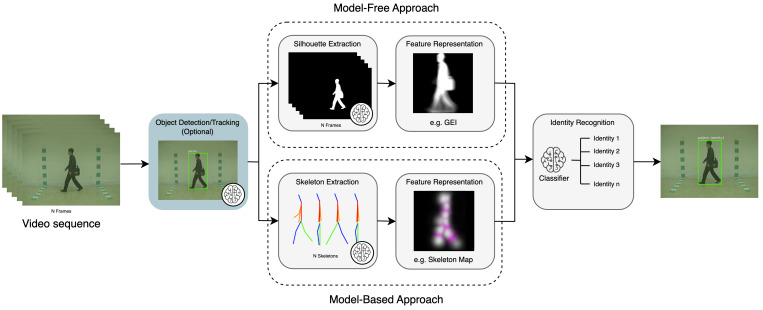
CVGR systems based on handcrafted representations typically employ one of two approaches: the systems extract silhouettes from 2D images (model-free) or rely on human body models (model-based). The video sample shown in the figure comes from the CASIA-B dataset [23].

In contrast to DL, handcrafted features are manually designed based on researchers’ domain expertise and understanding of distinctive gait characteristics. These features capture the unique structure, motion, and dynamics of an individual’s walking pattern. Handcrafted features can be categorised into two groups: model-based and model-free. Model-based approaches focus on extracting skeletal information and joint positions to capture the localised movement patterns of each body part [8]. Conversely, model-free methods (also known as appearance-based methods) directly extract features (primarily silhouettes) from a sequence of gait cycles [1]. Table 2 compares handcrafted-based and DL-based feature representations. Additionally, Figure 4 illustrates a common CVGR system, including two potential modalities for feature extraction: model-based and model-free. The following sections will provide a more detailed exploration of these modalities.

### 4.1. Model-Based Representations

Model-based gait recognition requires constructing a mathematical model that represents the human body and its movements during walking. This model can range from a simple stick figure to a complex biomechanical model incorporating muscle activations and joint constraints [8,9]. Other model-based approaches utilise specialised sensors (e.g., Kinect [26] and others mentioned in Section 3.3), or pre-trained models (e.g., OpenPose [38] and MediaPipe [39]) to create skeleton-like representations. By extracting spatial and temporal information from human body joints, this approach can distinguish one person’s gait from another’s. Consequently, they have gained significant attention in recent years for their ability to handle current CVGR challenges such as viewpoint variations, occlusions, and changes in clothing. Table 3 illustrates this rise in investigations of model-based approaches by comparing three important methods in this area.

#### 4.1.1. Pose Estimation

One prominent model-based technique is pose estimation, which involves detecting human joint positions in 2D or 3D space. This is achieved using 2D or 3D pose estimation models that detect key joints (e.g., hips, knees, and ankles) in each frame of a video sequence, forming a structured representation of human motion. To improve robustness, recent works employ pose normalisation techniques (e.g., centre and scale normalisation) that align body joints in a consistent frame of reference, reducing the variability caused by camera angles and distances [43]. The following is a sample formulation of pose estimation.

Let the human body pose at time step *t* be represented by a set of joints, $P_{t}=\left\{p_{1}^{t},p_{2}^{t},\cdots ,p_{J}^{t}\right\}$, where $p_{j}^{t}\in R^{2}$ or $R^{3}$ represents the 2D or 3D coordinates of the *j*-th joint at time *t*, and *J* is the total number of joints. For a sequence of poses across time, the full set of poses is given by $P=\{P_{1},P_{2},\cdots ,P_{T}\}$, where *T* is the total number of frames in the sequence. Thus, we can define the joint coordinate representation, where each joint $p_{j}^{t}$ at frame *t* is represented by its coordinates, depending on whether it is 3D or 2D pose estimation, as shown in Equation (1).

(1) $$p_{j}^{t}=\left[\begin{array}{c} x_{j}^{t}\\ y_{j}^{t}\\ z_{j}^{t} \end{array}\right]\;\mathrm{or}\;p_{j}^{t}=\left[\begin{array}{c} x_{j}^{t}\\ y_{j}^{t} \end{array}\right]$$

For model-based gait recognition, pose estimation models extract features from the set of joint coordinates. Typically, a feature extraction can be formulated as a function, *f*, applied to the sequence of joint positions, as defined in Equation (2), where $F\in R^{d}$ is a *d*-dimensional feature vector representing the gait. An important step in this methodology is normalisation, which aims to remove variations due to camera angles or distance. This is achieved as shown in Equation (3), where $p_{\mathrm{centre}}^{t}$ is the centre of the body (e.g., the hip joint), and $p_{\mathrm{height}}^{t}$ is the height of the person, used for scaling.

(2) $$F=f\left(P\right)=f\left(\{P_{1},P_{2},\cdots ,P_{T}\}\right)$$

(3) $$p_{j}^{t,\mathrm{norm}}=\frac{p_{j}^{t}-p_{\mathrm{centre}}^{t}}{∥p_{\mathrm{height}}^{t}∥}$$

Recent works on pose estimation-based gait recognition have achieved compelling results. For instance, the 3D skeleton-based PoseGait model, proposed by Lia et al. in 2020 [31], uses CNNs to extract multi-dimensional gait features from body joint trajectories. More recently, in 2023, Fu et al. introduced the novel framework GPGait [7] to address pose-based models’ main limitation: their inability to generalise across different scenarios. GPGait utilises two types of gait feature representations: human-oriented transformation (HOT) and human-oriented descriptors (HOD). HOT aligns human pose data from various camera viewpoints into a unified, stable representation, while HOD comprises multi-branch feature extraction modules that generate discriminative, domain-invariant features from this unified pose. Finally, to classify features, the authors employed a part-aware graph convolutional network (PAGCN), which efficiently implements graph partition and constructs local-global relationships through mask operations on the adjacency matrix.

#### 4.1.2. Skeleton Maps

Recent progress in skeleton maps for model-based gait recognition has focused on improving the precision and compactness of gait representations derived from human joint coordinates. Figure 4 illustrates an example of skeleton maps, which are used in SkeletonGait [43]. The authors of SkeletonGait generated heatmaps from joint coordinates, aiming to eliminate gait-unrelated information (e.g., walking trajectory and filming angle) through data normalisation techniques. This includes centre- and scale-normalisation to filter out irrelevant information like walking trajectory and camera distance, ensuring a cleaner representation of gait features. In summary, this approach further refines skeleton maps by leveraging only essential gait-related details, improving accuracy on challenging outdoor datasets like OUMVL [44] and GREW [16].

The representation of skeleton maps can be formulated as follows: Let the human skeleton at frame *t* be represented as a skeleton map, $S_{t}$. This map consists of a set of joint positions, $S_{t}=\left\{s_{1}^{t},s_{2}^{t},\ldots ,s_{J}^{t}\right\}$, where $s_{j}^{t}\in R^{2}\;\mathrm{or}\;R^{3}$ denotes the position of the *j*-th joint at frame *t*. The joint feature vector can then be defined as a collection, $s_{j}^{t}$, augmented with additional features such as velocity, $v_{j}^{t}$, and angles, $\theta_{j}^{t}$, at joint *j* at time *t*, as denoted next:

(4) $$h_{j}^{t}=\left[\begin{array}{c} x_{j}^{t}\\ y_{j}^{t}\\ z_{j}^{t}\\ v_{j}^{t}\\ \theta_{j}^{t} \end{array}\right]$$

Using joint feature vectors, we can construct the feature matrix for the skeleton map at frame *t*, as shown in Equation (5), where *d* is the dimension of the joint feature vector. This matrix effectively captures the spatial and temporal characteristics of human dynamics.

(5) $$H^{t}=\left[\begin{array}{c} h_{1}^{t}\\ h_{2}^{t}\\ \vdots \\ h_{J}^{t} \end{array}\right]\in R^{J\times d}$$

#### 4.1.3. Graph-Based Models

Graph-based models for model-based gait recognition have seen significant advancements in recent years, primarily leveraging graph convolutional networks (GCNs) to better capture spatial and temporal dependencies in human motion. For instance, in 2023, GaitGraph [40] and its successor, GaitGraph2 [41], treated human joints as nodes and their connections (limbs) as edges, processing these data to capture both local and global spatial relationships. This approach enables the capture of both local movements (e.g., individual limb movements) and global motion patterns (e.g., full-body gait dynamics) crucial for distinguishing gaits across individuals. These models have demonstrated improved robustness across various datasets and under different environmental conditions, particularly in handling occlusion, different viewing angles, and variations in walking surfaces.

The mathematical representation of a graph-based model for gait recognition can be formulated as follows. A human skeleton at frame *t* can be represented as a graph $G_{t}=\left(V,E\right)$, where $V=\{v_{1},v_{2},\cdots ,v_{J}\}$ is the set of *J* joints (nodes), and E⊆V×V is the set of edges representing the physical connections between joints (limbs). Each node $v_{j}$ at frame *t* is associated with a feature vector, $h_{j}^{t}\in R^{d}$, representing the position (e.g., 2D or 3D coordinates) and possibly other information, such as velocity or joint angles:

(6) $$h_{j}^{t}=\left[\begin{array}{c} x_{j}^{t}\\ y_{j}^{t}\\ z_{j}^{t} \end{array}\right]\;(\mathrm{for}\;3D\;\mathrm{pose}).$$

Thus, the feature matrix for the entire skeleton at frame *t* is as follows:

(7) $$H^{t}=\left[\begin{array}{c} h_{1}^{t}\\ h_{2}^{t}\\ \vdots \\ h_{J}^{t} \end{array}\right]\in R^{J\times d}$$

The connections between joints (limbs) are captured via the adjacency matrix $A\in R^{J\times J}$, where the following applies:

(8) $$A_{ij}=\left\{\begin{array}{cc} 1 & \mathrm{if}\;\mathrm{there}\;\mathrm{is}\;\mathrm{an}\;\mathrm{edge}\;\mathrm{between}\;\mathrm{joints}\;v_{i}\;\mathrm{and}\;v_{j},\\ 0 & \mathrm{otherwise}. \end{array}\right.$$

### 4.2. Model-Free-Based Representations

Model-free representation methods for gait recognition, also known as appearance-based gait recognition, extract features directly from gait data without relying on explicit models of the human body or specific anatomical configurations. This direct extraction makes them practical for 2D gait data and particularly well suited for edge computing due to their efficiency in online processing pipelines. However, it is important to highlight the decreasing trend in research on these types of representations due to the rise of model-based methods and deep learning.

#### 4.2.1. Motion Energy Images and Motion History Images

Human motion analysis has long been an area of interest for computer vision researchers. Bobick and Davis [45] proposed two of the initial feature representations: the motion-energy image (MEI) and the motion-history image (MHI). An MEI is a binary image that highlights where motion has occurred within a certain time window, with pixels taking a value of 1 to indicate motion and 0 to indicate no motion. Equation (9) defines this, where D(x,y,t) represents a binary image sequence indicating regions of motion, with *x* and *y* as the spatial coordinates for frame *t* and τ as the temporal extent of the motion.

(9) $$E_{\tau }\left(x,y,t\right)=\overset{\tau -1}{\underset{i=0}{⋃}}D\left(x,y,t-i\right)$$

Conversely, the MHI aims to produce a scalar-valued image where more recently moving pixels appear brighter. Equation (10) defines the MHI, where $H_{tau}$ is a function representing the temporal history of motion at a specific pixel. Inspired by the MHI, Lam and Lee proposed a derived gait representation called the motion silhouettes image (MSI) [46]. In their proposal, they replaced τ with 255, and they made the MSI simpler and easier to implement, maintaining most of the functionality of the MHI.

(10) $$H_{\tau }\left(x,y,t\right)=\left\{\begin{array}{cc} \tau & \mathrm{if}\;D(x,y,t)=1\\ max(0,H_{\tau }\left(x,y,t-1\right)-1) & \mathrm{otherwise} \end{array}\right.$$

#### 4.2.2. Gait Energy Images and Gait History Images

Gait energy images (GEIs) are 2D representations of a person’s gait over time, capturing their movement into a single composite image [47]. They are often created by averaging or accumulating a series of aligned silhouettes, motion images (such as MSIs), or depth images. As shown in Figure 4, a GEI provides a holistic view of an individual’s walking style, capturing key characteristics like stride length, arm swing, and body posture within a single image, while preserving all temporal information. Equation (11) shows the formulation of a GEI, where $B_{t}\left(x,y\right)$ represents a binary gait silhouette image at frame *t*, and *n* is the total number of frames within the cycle. While the GEI captures both dynamic (movement-related) and static (shape-related) features from the gait sequence, it lacks the ability to characterise the specific timing of foreground activity at each pixel. To address this, an improved temporal feature representation method called the gait history image (GHI) was proposed by Liu and Zheng [48]. The GHI is designed to represent not only static and dynamic characteristics but also the spatial and temporal variations within a gait cycle. Equation (12) defines this, where S(x,y) represents static pixels, calculated by taking the intersection of all motion detection images D(x,y,t) from time 1 to τ.

(11) $$G\left(x,y\right)=\frac{1}{n}\sum_{t=1}^{n}B_{t}\left(x,y\right)$$

(12) $$E_{GHI}\left(x,y\right)=\left\{\begin{array}{cc} \tau & \mathrm{if}\;S(x,y)=1\\ \sum_{t=1}^{t}D\left(x,y,t\right)\cdot \left(t-1\right) & \mathrm{otherwise} \end{array}\right.$$

Both GHIs and GEIs are widely used methods in modern gait recognition workflows. However, their improvement remains an active area of research. For example, Zebhi et al. [49] proposed combining GHIs with their new descriptors named time-sliced averaged gradient boundary magnitude (TAGBM) to abstract spatial and temporal information from videos into templates for human activity recognition. Similarly, Wang et al. [50] recently presented DyGait, a CVGR system that improves traditional GEIs. They introduced a dynamic augmentation module (DAM) that enhances the traditional GEI approach by focusing on the dynamic aspects of human gait. While traditional GEIs primarily capture static body parts, like the torso, the DyGait method augments this by emphasising the dynamic movements of limbs, allowing for more robust gait recognition, especially in challenging conditions like changes in clothing or when individuals are carrying items.

#### 4.2.3. Gait Entropy Images

Unlike GEIs, gait entropy images (GEnIs) focus on the dynamic elements of gait by encoding the randomness of pixel values within the silhouette over a complete gait cycle [51]. GEnIs are constructed by calculating the Shannon entropy (defined in Equation (13)) for each pixel over a complete gait cycle. This means that pixels with high values in the GEnIs correspond to body parts with high movement variability, such as the arms and legs.

(13) $$H\left(x,y\right)=-\sum_{k=1}^{k_{n}}p_{k}\left(x,y\right)\log_{2}p_{k}\left(x,y\right)$$

In the previous equation, $p_{k}\left(x,y\right)$ is the probability that the pixel at the coordinates x,y takes on the $k^{th}$ value. For binary images, there are only two possible intensity values, so $k_{n}=2$. Therefore, the gait entropy image G(x,y) can be calculated in the function of a scaled and discretised H(x,y), obtained as follows:

(14) $$G\left(x,y\right)=\frac{\left(H\left(x,y\right)-H_{min}\right)\times 255}{H_{max}-H_{min}}$$

where $H_{min}$ represents the minimum value of H(x,y), and $H_{max}$ represents the maximum value. The resulting frame will highlight the dynamic areas of the human body with higher intensity values, while static parts will have lower intensity values.

#### 4.2.4. Gait Flow Image

A gait flow image (GFI) aims to capture the dynamic motion information in a gait sequence by utilising optical flow [52]. Optical flow algorithms estimate the apparent motion of pixels between consecutive frames in a video sequence. For gait movement patterns, this provides a compelling means to capture the subtle dynamics of human movement, such as the swinging of limbs, the shifting of body weight, and the overall rhythm of walking. A GFI is constructed by first extracting silhouettes of the walking person from a sequence of images and then calculating the optical flow between consecutive frames in the silhouette sequence. The primary formula involved in calculating optical flow is the optical flow constraint, defined as follows:

(15) $$I_{x}u+I_{y}v+I_{t}=0$$

In this equation, $I_{x}$ represents the spatial derivative of the image intensity in the x-direction (horizontal), $I_{y}$ represents the spatial derivative of the image intensity in the y-direction (vertical), and $I_{t}$ represents a temporal derivative of the image intensity (change in intensity over time). Furthermore, *u* and *v* represent the horizontal and vertical components of the optical flow vector, respectively.

Optical flow qualities were recently explored by Ye et al. [53], who implemented the gait optical flow image (GOFI)—a subtype of the GFI in which the optical flow information is directly encoded into the image from RGB frames—to add instantaneous motion direction and intensity to original gait silhouettes. They then implemented a neural network called the Gait Optical Flow Network (GOFN), which allows for a more detailed and robust analysis of gait patterns. Despite its qualities, optical flow requires high computational power to process high-resolution videos or for real-time applications, which can limit the scalability and efficiency of gait recognition systems, particularly on resource-constrained devices [8].

#### 4.2.5. Other Model-Free Representation Methods

This section summarises other noteworthy mode-free gait representations. Additionally, Table 4 compares all the feature representations introduced in this section.

- To address the issue of poor image segmentation in gait recognition frameworks, Chen et al. [54] proposed the frame difference energy image (FDEI) representation. This is a robust representation designed to mitigate the impact of incomplete silhouettes.
- Wang et al. [55] proposed the chrono-gait image (CGI), which encodes the temporal information by assigning different colours to the silhouettes based on their position in the gait cycle, generating a single CGI with richer information.
- To tackle the issue of variations in clothing and carried objects during walking, Zhang et al. [56] proposed the active energy image (AEI) representation. This approach focuses on dynamic body parts, discarding static ones, by calculating the difference between consecutive silhouettes in a gait sequence.
- He et al. [57] proposed the period energy image (PEI), a multichannel gait template designed as a generalisation of GEI. It aims to enrich spatial and temporal information in cross-view gait recognition, maintaining more of this information compared to other templates.
- The frame-by-frame gait energy image (ff-GEI) presented in [58] effectively expresses available gait data, relaxes the gait cycle segmentation constraints imposed by existing algorithms, and is better suited to the requirements of DL models.

**Table 4:** Comparison of model-free gait feature representations, sorted by year of publication. Note the decline in the proposal of compelling model-free representations since 2020, though previous representations remain in use.

Gait Feature Representation	Year	Pros	Cons	Frequency of Use	Recent Applications
ff-GEI [58]	2020	Captures energy per frame, useful for detailed gait dynamics.	Computationally expensive, large data requirements.	Rare	No recent works found.
PEI [57]	2019	Highlights periodic gait motion, useful for recognising consistent gait patterns.	Sensitive to changes in walking speed and conditions.	Less frequent	[59]
GFI [52]	2011	Captures velocity and flow of movement, sensitive to gait dynamics.	Computationally complex, sensitive to noise and illumination changes.	Less frequent	[8,53]
CGI [55]	2010	Combines motion with temporal encoding, captures both spatial and temporal information.	Computationally complex, sensitive to frame rate and noise.	Less frequent	No recent works found.
AEI [56]	2010	Captures active energy regions, good for detecting dynamic motion.	Complex to compute, requires high-quality input for effectiveness.	Less frequent	[60]
FDEI [54]	2009	Highlights regions of change between frames, simple to compute.	Misses subtle motions, highly sensitive to noise.	Moderately frequent.	[61]
GEnI [51]	2009	Encodes gait variability and randomness, useful for capturing subtle dynamics.	Sensitive to noise, more complex to compute.	Less frequent	[62]
GHI [48]	2007	Captures both spatial and temporal aspects of movement.	More computationally expensive, sensitive to noise.	Less frequent	[49]
GEI [47]	2006	Robust to clothing and carrying conditions, captures averaged body silhouettes.	Loses fine temporal details, less effective in occlusion scenarios.	Very frequent	[50]
MSI [46]	2005	Simpler and easier to implement than MHI.	Primarily focuses on shape information without incorporating the temporal dynamics of the gait.	Moderately frequent	No recent works found.
MEI [45]	2001	Simple and efficient, captures where motion has occurred.	Lacks detailed temporal motion information, sensitive to noise.	Moderately frequent	No recent works found.
MHI [45]	2001	Captures temporal motion patterns, simple to compute, compact representation.	Sensitive to noise, cannot capture subtle variations in movement.	Less frequent	No recent works found.

## 5. Gait Representation Dimensionality Reduction

Dimensionality reduction (DR) techniques play a vital role in gait recognition by eliminating redundant or irrelevant features from gait representations. This minimisation process offers several benefits: it reduces computational costs, filters out noise, and improves the accuracy of subject recognition [3]. While DL encoders are now commonly used for DR, the resulting feature representations for gait sequences can still be quite high-dimensional. To address this, DR methods can reduce the number of features, making subsequent classification or recognition tasks more manageable [63]. This is particularly important because processing high-dimensional features can be computationally demanding, especially for real-time applications or resource-constrained devices. Therefore, DR helps alleviate this burden, enabling efficient edge computing.

### 5.1. Linear Techniques

One of the most commonly used DR techniques in gait recognition is principal component analysis (PCA) [64,65]. PCA aims to identify the optimal projection that maximises the variance of the information, effectively reducing the dimensionality of feature vectors. This technique compresses large data representations into smaller feature sets that still retain the crucial information of the original representation [66]. For instance, in the framework proposed by Gupta et al. [67], a boundary energy image (BEI) served as the feature representation. PCA was then applied for DR before a linear discriminant analysis (LDA) algorithm was used for gait classification.

Regarding LDA, its main goal is to find a new set of axes (linear combinations of the original features) that maximise the separation between different classes while minimising the variance within each class. This transformed space, with fewer dimensions, makes it easier to classify new data points. LDA has been applied in numerous CVGR frameworks. For example, Guo et al. [68] implemented LDA to the feature data reduction alongside a Gabor filter for feature extraction from GEIs and used an extreme learning machine algorithm for recognition and classification. Additionally, Wang et al. [69] introduced a generalised LDA based on the Euclidean norm (ELDA) to address the challenge of indistinguishability resulting from overlapping data in LDA, and they employed multi-class SVMs for gait classification. Furthermore, in [70], the authors presented a DR framework based on LDA and PCA in conjunction with a GEI representation and a cyclic recognition code (CRC) classification scheme for video-based gait recognition.

### 5.2. Non-Linear Techniques

Non-linear techniques for gait representation DR, such as Isomap [23], locally linear embedding (LLE) [71], and Laplacian eigenmaps [72], operate under the assumption that gait data exist on a low-dimensional manifold embedded in a high-dimensional space. These techniques aim to preserve the local and global structure of gait data while reducing their dimensionality. Moreover, t-distributed stochastic neighbor embedding (t-SNE) is another valuable DR technique commonly used for visualising high-dimensional data. By focusing on preserving local relationships between data points, it is useful for exploratory data analysis and identifying clusters in gait recognition tasks. For example, Che and Kong [73] investigated how t-SNE can be applied to GEI images of walking subjects after the images underwent discrete wavelet decomposition (DWT) to reduce their dimensionality. Their results demonstrated that this proposed feature extraction method effectively reduces computational complexity and preserves image information, leading to improved precision metrics when an SVM model is used.

Although non-linear techniques have shown promise in gait recognition, they are less frequently used in state-of-the-art DR for gait representation compared to linear techniques. This is primarily because most non-linear techniques can be computationally more expensive than linear methods, especially when dealing with large datasets. Moreover, this high computational cost can significantly limit their implementation on embedded devices. However, manifold learning techniques may still offer advantages when data exhibit strong non-linear relationships or when interpretability is crucial [74].

### 5.3. Other Dimensionality Reduction Methods for Gait Recognition

The discrete cosine transform (DCT) is a widely used technique for DR in image-based gait recognition. It retains crucial gait features, such as body posture and limb movement, by utilising a limited set of low-frequency DCT coefficients. Prior research has demonstrated the effectiveness of DCT in reducing image dimensionality before classification tasks, where a significant number of DCT coefficients can be discarded, substantially decreasing the search space dimensionality [75]. In the context of gait recognition, Fan et al. [76] proposed an approach to DR that combines DCT and LDA. This method effectively leverages the frequency-domain information of the GEI representation to achieve higher recognition rates than other reviewed methods. In another study by Chhatrala et al. [77], the authors combined the Gabor function and DCT to extract features from gait representations, resulting in the extraction of highly discriminative components that led to high recognition accuracy.

The combination of two or more DR methods for gait recognition is also prevalent in the literature. Similarly to Che et al. [73], who combined t-SNE with DWT, Shivani and Singh [78] proposed a method for reducing redundant data and extracting features by combining DWT and DCT prior to classification. This approach effectively extracts critical features from gait images and video frames. Furthermore, Wen and Wang [79] introduced a gait recognition technique using sparse linear subspace to reduce the dimensionality of ff-GEIs generated from primary gait features. This approach was tested on two well-known gait datasets, and it demonstrated good performance.

## 6. Classification of Gait Feature Representations

The final stage in gait recognition involves classifying the previously extracted and reduced gait feature representations. This section reviews the various classifiers commonly employed in this process.

### 6.1. Traditional Models

#### 6.1.1. Distance-Based Classification

Euclidean distance, Manhattan distance, or cosine similarity (defined in Equations (16), (17) and (18), respectively) are simple and widely used methods for classifying gait features in CVGR systems [80,81,82]. They help compare and match gait representations by calculating the distance between them. Another popular machine learning-based approach that leverages metrics is the k-nearest neighbours (k-NN) algorithm. This classifies a new gait sample based on the majority class among its ‘k’ nearest neighbours in the feature space. Neighbouring samples are defined using metrics such as Euclidean distance, Manhattan distance, or cosine similarity. Several studies have successfully employed k-NN in gait recognition. For example, Pratama et al. [83] combined a k-NN classifier with GEI, Gabor wavelets, and PCA. Additionally, Premalatha and Chandramani [84] utilised k-NN classification along with GEI partitioning and region selection.

(16) $$d\left(p,q\right)=\sqrt{\sum_{i=1}^{n}{\left(q_{i}-p_{i}\right)}^{2}}$$

(17) $$d\left(p,q\right)=\sum_{i=1}^{n}\left|q_{i}-p_{i}\right|$$

(18) $$\cos\left(p,q\right)=\frac{p\cdot q}{∥p∥∥q∥}$$

#### 6.1.2. Machine Learning

Traditional machine learning (ML) techniques for CVGR systems encompass a diverse set of models, among which k-NN and support vector machines (SVMs) have been two particularly popular choices for classification. The latter, SVMs, when applied to gait recognition, aim to map gait representations to a high-dimensional feature space where they can be effectively categorised [85]. Several notable contributions using SVMs for CVGR can be highlighted. For instance, Wattanapanich et al. [64] proposed a view-invariant gait recognition framework that utilised a One-Against-All SVM classifier. This framework sought to achieve robustness and address the challenges posed by unknown camera view angles and appearance changes. Their results demonstrated the framework’s robustness under varying camera view angles. In another study, Wang et al. [86] presented a gait recognition algorithm employing a multi-class SVM. This algorithm leveraged Gabor wavelets for gait feature extraction and two-directional, two-dimensional PCA ((2D) ^2^ PCA) for dimensionality reduction.

Moreover, hidden Markov models (HMMs) are prominent in gait analysis due to their ability to model the sequential and temporal dynamics of gait data, capturing variations in the gait cycle over time [87]. These traditional methods, including LDA [67,88] and Bayesian classifiers [89,90], often perform well with model-free features and have laid the groundwork for the DL techniques that now dominate the field. In contrast, model-based features often involve more complex data, such as joint angles, bone lengths, or motion parameters. This can lead to higher dimensionality, potentially making it harder for traditional models discriminate effectively [91].

### 6.2. Deep Learning

**Figure 5:**
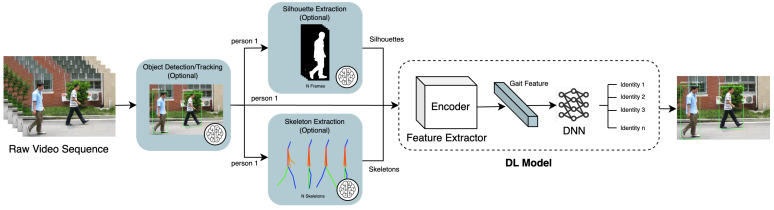
DL-based end-to-end gait recognition scheme for CVGR systems. The sample with the walking subjects shown in the figure comes from the Penn–Fudan dataset [94].

Deep learning (DL), a subfield of ML, utilizes artificial neural networks (ANNs) to learn complex patterns from data. ANNs with multiple layers, also known as deep neural networks (DNNs), have proven effective in tackling CVGR limitations such as intra-class variations (e.g., subjects wearing different clothes) [92] and cross-view [93]. CVGR pipelines based on DL can be categorized into two groups: end-to-end and hybrid. In end-to-end systems, the encoder component of DL models extracts subtle gait features, while the DNN deciphers previously elusive complex patterns. Figure 5 illustrates this, showing that a DL model can be fed with raw video data, frames containing silhouettes, or skeletons modelling the human body of a walking subject. While the gait sequence of a single person can be obtained using object detectors, segmentation algorithms, or model-based approaches (marked with brains in Figure 5), the end-to-end approach focuses on training a single model to handle the entire gait recognition task.

In contrast to end-to-end systems, hybrid pipelines might also include handcrafted feature extraction and dimensionality reduction modules (detailed in Section 4 and Section 5, respectively). Figure 4 shows how these tasks fit into the traditional gait recognition pipeline, with brain symbols marking tasks suitable to be performed using individual DL models. Furthermore, hybrid pipelines can incorporate object detection and tracking algorithms to first recognise multiple subjects. The following sections explore representative DL models used frequently in CVGR systems.

#### 6.2.1. Convolutional Neural Networks

CNNs are a specialised type of DNN designed primarily for processing and analysing visual imagery. Inspired by the visual cortex in humans, CNNs employ 2D convolutional operations to extract features, progressing from coarse-grained to fine-grained details. These extracted features are then passed to a neural network module for final inference. Notably, 3D convolutional layers can also be used to extract gait features from sequential imagery, such as GEI sequences [95] or 3D volumes representing body parts [96].

A key advantage of CNNs is their ability to recognise patterns irrespective of where they appear in an image [97]. This characteristic made them particularly valuable for both model-free [59,62] and model-based [98] approaches. As illustrated in Figure 1b, CNNs are amongst the most frequently used models for CVGR systems. The following section presents some compelling works applying CNNs to gait recognition:

- In 2022, Ambika et al. [99] proposed an approach based on a CNN-MLP (Multilayer Perceptron) for gait classification, aiming to be robust to subjects’ velocity variations and appearance covariates such as carrying a backpack.
- Researchers in [96] (2021) and [100] (2023) proposed CNN-based methods to split vertically the gait sequence into multiple parts and extract sequential, hierarchical gait features using 3D convolutional layers. Both methods achieved state-of-the-art results, demonstrating the effectiveness of CNNs for gait feature extraction.
- Further CNN-based gait recognition approaches were proposed in [59,98,101,102].

#### 6.2.2. Autoencoders

An autoencoder is a type of ANN that consists of an encoder and a decoder, with the primary goal of reconstructing its input as accurately as possible using the decoder module [103]. Both encoder and decoder modules can have convolutional or deconvolutional layers to better process visual imagery, and the bottleneck layer (middle layer) typically serves as a dimensionality-reduced representation. Autoencoders have been utilised in model-free pipelines to address appearance change issues like varying view angles [104] and for completing missing parts in GEI representations [105]. On the other hand, recent compelling investigations for model-based methods include the following:

- In 2023, Guo et al. [106] introduced a physics-augmented autoencoder (PAA) that integrates a physics-based decoder. By incorporating physics, the learned 3D skeleton representations become more compact and physically plausible.
- In the same year, Li et al. [107] proposed a novel gait recognition method that leverages the Koopman operator theory and invertible autoencoders to improve interpretability and reduce the computational cost of gait recognition by learning a low-dimensional, physically meaningful representation of gait dynamics that captures the complex kinematic features of gait cycles.

#### 6.2.3. Generative Adversarial Networks

Similarly to autoencoders, generative adversarial networks (GANs) are a powerful DL approach capable of producing new data that closely resemble the training data. In the context of gait recognition, various GAN approaches have been adopted to address view variations or appearance changes. For example, consider the following:

- Yu et al. [108] proposed a GAN to tackle gait recognition limitations related to appearance changes by generating realistic-looking gait images. The results demonstrated excellent performance on large datasets, where a canonical side-view gait image was generated without needing prior knowledge of the subject’s view angle or clothing.
- To further research the challenge of view variations in gait recognition, Zhang et al. [109] proposed the View Transformation GAN (VT-GAN). This model translates gaits between any two views using an identity preserver module to prevent the loss of personal identity information during transformations.

#### 6.2.4. Capsule Neural Networks

Capsule networks, or CapsNets, are a type of DNN designed to address some limitations of traditional CNNs, particularly in understanding hierarchical relationships and spatial information within images [110]. Capsules, the fundamental building blocks of CapsNets, are groups of neurons that encapsulate information about specific features or entities in the input data. Specifically, each capsule in a CapsNet comprises a set of neurons whose outputs represent different attributes of a single feature. CapsNets have been successfully used in gait recognition to mitigate performance drops when dealing with various walking conditions or multiple viewing angles. For example, Sepas-Moghaddam et al. [111] addressed the gait recognition covariate problem by proposing a DNN for learning to transfer multiscale partial gait representations. This network employs CapsNets to learn deeper part-whole relationships and assign more weight to highly relevant features while eliminating spurious dimensions. The proposed model consists of a partial feature extraction stage for gait maps, a BGRU layer, a capsule attention module, and a final layer with a softmax activation function for classification. The model demonstrated impressive rank-1 recognition results, achieving mean accuracy values of 95.7% for normal walking, 90.7% for carrying a bag, and 72.4% for varying clothing conditions. Other works, such as [112,113,114], have also incorporated capsule modules into their gait recognition approaches.

#### 6.2.5. Recurrent Neural Networks

RNNs, an extension of DNNs well suited to processing sequential data, offer another approach to gait recognition classification [115]. These models, along with long short-term memory (LSTM) models—a type of RNN adept at learning order dependency in sequence prediction—have been widely used for gait recognition by leveraging their inherent ability to capture temporal dependencies [98]. Although RNNs and LSTMs seem like a natural fit for gait recognition due to their ability to handle sequences, they have largely been overshadowed by CNNs, primarily because of their computational cost and limitations in capturing spatial details [8,63]. This is particularly relevant for resource-constrained environments or applications demanding real-time performance, where RNNs/LSTMs might not be the most practical choice [116,117]. Nevertheless, they can still be valuable when capturing temporal dynamics is paramount, especially when combined with CNNs. Some remarkable works include the following:

- Wang and Yan [58] combined LSTM with convolutional layers (namely ConvLSTM) and trained the resulting model using ff-GEIs. Their method outperformed several state-of-the-art methods in multi-view angle gait recognition.
- To address the challenge of occlusions in gait recognition, Sepas-Moghaddam et al. [118] proposed a method for learning invariant convolutional gait energy maps with an attention-based recurrent model. Their network structure utilised partial representations by decomposing learned gait representations into convolutional energy maps. A recurrent learning module composed of bidirectional gated recurrent units (BGRU) was then used to exploit relationships between these partial spatiotemporal representations, coupled with an attention mechanism to focus on crucial information.

#### 6.2.6. Graph Neural Networks

Graph neural networks (GNNs) are a class of DL models designed to process data that can be represented as graphs. As explained in Section 4.1.3, gait features (e.g., skeleton maps) can also be represented as graphs, making it possible to combine them with GNNs for efficient gait recognition. Unlike traditional ANNs, which typically operate on structured data (like grids or sequences), GNNs can naturally handle non-Euclidean data by aggregating and passing information between neighbouring nodes. This allows GNNs to capture complex relational structures and dependencies within the gait feature data [40]. Crucially, GNNs have been shown to be capable of running on edge devices with appropriate optimisation techniques listed in Section 8 [119].

Graph convolutional networks, a subtype of GNNs, are designed to process graph data by applying convolutions. In gait recognition, GCNs can capture the complex relationships between joints in a gait sequence. Both GNNs and GCNs have recently shown great promise by modelling the structural and temporal dependencies of human body joints and keypoints for gait recognition. For example, Guo et al. [120] proposed a notable work in this field that uses a spatial–temporal graph convolutional network (ST-GCN) to capture both spatial joint relationships and temporal walking dynamics. These and other aforementioned GNN-based approaches, such as [7,41], have successfully leveraged the inherent structural and dynamic properties of human gait, leading to state-of-the-art results on benchmark datasets like CASIA-B [23] and OU-MVLP [44].

Recently, GNNs have also obtained meaningful results with 3D and 4D point clouds. For example, Ma et al. [121], proposed the Dynamic Aggregation Network (DANet), a network based on the Dynamic Graph CNN (DGCNN) [122]. DANet operates on dynamic graph structures and uses edge convolution to learn both the local and global structure of the point cloud, which is crucial for gait recognition from dynamic 3D data. Specifically, DANet dynamically learns local and global motion patterns from gait sequences, enhancing the model’s ability to focus on key regions within the gait data and achieving compelling results on datasets such as Gait3D [5].

#### 6.2.7. Transformers and Attention Mechanisms

Transformer networks excel at capturing long-range dependencies in sequential data, and they are effective at processing gait in its different forms, for which temporal variations are key [123]. Furthermore, attention mechanisms enable transformers to focus on specific parts of complex inputs, thereby increasing their performance. Some compelling works in this field include the following:

- To enhance discrimination between different classes of gait features, Wang and Yan [124] (2021) presented a self-attention-based gait classification model that combines non-local and regionalised features. This combination helps identify relevant non-local features, which are then refined by a two-channel network.
- Jia et al. [125] (2021) proposed a CNN joint attention mechanism (CJAM) for identifying the most crucial pixels in a gait sequence. Their workflow uses a CNN to extract feature vectors from the initial gait frames and feeds them into an attention model comprising encoder and decoder layers, followed by linear operations for information transformation and a softmax function for classification. Twelve experiments demonstrated that this attention model outperforms others in terms of reducing errors, with the CJAM model achieving accuracy improvements of 8.44%, 2.94%, and 1.45% over 3D-CNN, CNN-LSTM, and a simple CNN, respectively.
- Mogan et al. [126] (2022) converted GEI representations into 2D flattened patches and passed them into a vision transformer model consisting of an embedding layer (with patch embedding applied to the sequence of patches), a transformer encoder for achieving a final representation, and a multi-layer perceptron that performs the classification based on the first token of the sequence. Experiments on small and large datasets demonstrated the scalability of the vision transformer model, showcasing its robustness against noisy and incomplete silhouettes and its remarkable subject identification capability regardless of the different covariates. In 2023, the same team proposed assembling a similar Transformer model with two other architectures: a DenseNet-201 and a VGG-16 [127]. The experimental results showed substantial improvement using the CASIA-B [23] and OU-ISIR [18] datasets.
- Li et al. [123] (2023) proposed TransGait, a novel gait recognition framework that utilises a transformer to effectively fuse silhouette and pose information, leveraging the strengths of both feature representations.
- Researchers have also recently leveraged self-attention [126] and spatial–temporal attention [128] to improve the discriminative capacities of CVGR systems.

In summary, the CVGR field is actively exploring novel models to enhance performance in challenging real-world scenarios. However, model complexity can hinder deployment on low-resource devices, often necessitating cloud-based infrastructure. For instance, although deploying transformers on embedded devices is possible [129], they can be quite resource-intensive, demanding substantial memory for parameters and intermediate computations. Similarly, the complex computations of other aforementioned DL models often require significant processing power. Table 5 further illustrates this by comparing the suitability of different DL models for edge computing.

## 7. Edge-Oriented Inference Architectures

**Figure 6:**
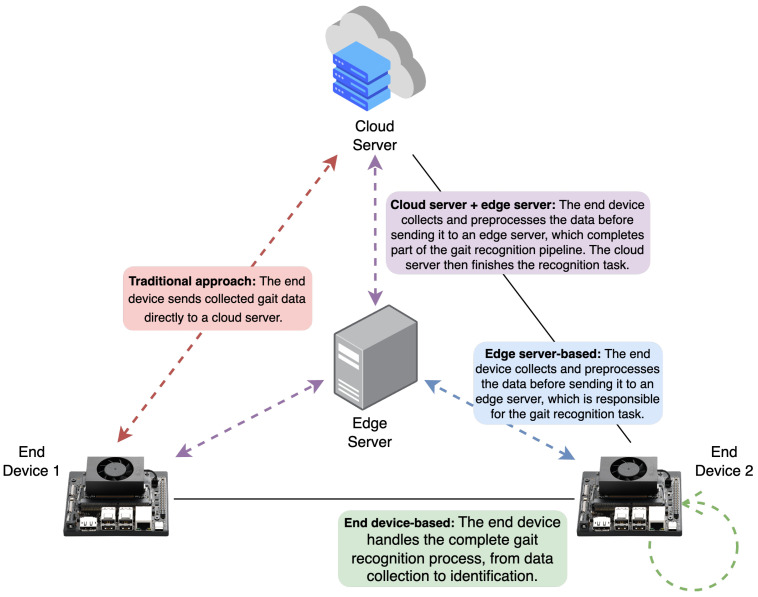
Graphical depictions of various edge-oriented inference architectures.

**Figure 7:**
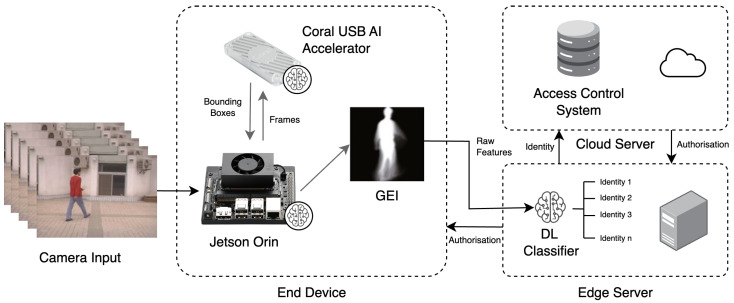
A large-scale scalable framework to support gait recognition computations in a distributed manner. This framework would incorporate multiple nodes and an edge server to handle data acquisition, detection, segmentation, and classification, enabling more feasible real-time computation. The video sample shown in the figure comes from the CASIA-A dataset [24].

Edge computing is rapidly evolving and gaining traction across industries. Advancements in hardware, software, and AI algorithms are making intelligent devices increasingly powerful, allowing for the deployment of more sophisticated AI applications directly at the edge. This trend, also known as edge AI, is expected to continue, leading to wider adoption of intelligent systems in the years to come [137]. Within this context, three main strategies for edge computing deployment can be observed in the current literature: edge server-based, end device-based, and a hybrid approach combining an edge server + end devices or an edge server + a cloud server (refer to Figure 6 for a visual representation). As shown in Figure 7, these approaches are not independent of each other and can be combined simultaneously to enable more feasible real-time computation in CVGR systems. In this figure, an edge computing development card (a small, specialised computer designed to bring AI and other processing capabilities directly to the edge) and a USB AI accelerator (a small device that plugs into the USB port of a computer or other system to provide dedicated hardware to accelerate inference) would perform different tasks necessary for the CVGR pipeline. The following sections will describe these strategies in detail.

### 7.1. Edge Server-Based

Unlike cloud-based servers, edge servers are located at the “edge” of a network, close to where data are collected. They vary in form factor, ranging from powerful rack-mounted servers to high-capability desktop computers. In this model, end devices collect data and transmit them directly to an edge server, which processes the input and sends the results back. The inference pipeline on the edge server may include one or more steps for pre-processing, recognising, and post-processing visual data, and the server must be able to handle visual data from multiple end devices simultaneously. However, a primary drawback of this model is its reliance on network bandwidth; processing cannot occur without a local area network. Galanopoulos et al. [138] provide an example of this approach, where mobile devices were programmed to collect video data and send it to an edge server for real-time processing and analytics.

### 7.2. End Device-Based

End device-based deployment refers to a system architecture where the entire processing pipeline, from data capture to inference, runs directly on the end device. This approach significantly enhances user privacy and reduces latency, which are crucial factors for CVGR systems. However, end devices often have limited processing power and memory. Although CVGR systems can execute on laptops or desktops with powerful graphics processing unit (GPU) chips, implementing them on less expensive hardware is possible. Edge computing development cards and USB AI acceleration devices with GPU, tensor processing unit (TPU), and/or vision processing unit (VPU) chips (detailed in Table 6 and Table 7, respectively) offer a low-cost solution for deploying CVGR as an embedded system. Additionally, compression tools (e.g., TensorFlow Lite, TensorRT, OpenVINO, and ONNX) and model optimisation techniques (e.g., knowledge distillation, model design, and quantisation) can be applied for enhanced deployment. Section 8 further discusses optimisation strategies based on these techniques.

DL-based systems deployed directly on end devices have found applications in diverse fields, ranging from medical devices that use infrared lighting to locate veins [139] and indoor systems for potential airborne disease infection monitoring [140] to unmanned surface vehicles for aquatic weed removal [141]. In the realm of CVGR systems, a significant contribution was made by Tiñini et al. [131], who proposed one of the first fully embedded CVGR systems. The authors used a Jetson Nano Development Card equipped with an OAK-D camera. Their approach leveraged a pre-trained person detection model, MobileNetv2, deployed on the OAK-D device, with subsequent representation extraction using a U-Net and inference performed on the Jetson Nano with a CNN. Their proposed system achieved an impressive inference time of 35.8 milliseconds per gait representation recognition.

### 7.3. Edge Server + End Devices

When deploying the entire CVGR pipeline on an end device is impractical due to limited memory and energy resources, partitioning a computer vision pipeline or model is possible. This involves keeping some parts on an edge server and deploying others on end devices. Factors like network latency, memory footprint, and energy consumption can help determine the optimal number of model splits and the execution location of each part. However, the final deployment strategy should ideally be based on experimentation, empirical criteria, and actual performance evaluation. Neurosurgeon, a scheduling strategy presented in [142], offers a compelling proposal to automate decisions on where to split a model and how to deploy its portions. Another example of this hybrid approach is found in [143], where armed robbery detection is achieved by combining YOLO-based weapon detectors on end devices with a CNN deployed on an edge server. The edge server then sends notifications to homeowners only if anomalies are confirmed by both the end devices and itself, thus reducing the false positive rate.

### 7.4. Edge Server + Cloud Server

Also known as edge–cloud, this approach aims to balance computing tasks between cloud servers and edge servers when the latter’s capabilities are insufficient. This is particularly valuable now that every data instance is crucial and can contribute to the generalisation of DL models. For example, an edge server can collect and process individual samples sent from end devices, with these data being synchronised with a cloud server once the edge server reaches its memory capacity. This hybrid approach can address the limitations of traditional cloud-based approaches for video analysis, which often suffer from prohibitive bandwidth consumption and high response latency. For example, Han et al. [144] proposed ECCVideo, an edge–cloud collaborative video analysis system that focuses on low-latency applications by efficiently utilising resources at both the edge and in the cloud. This approach has proven successful in scenarios where the capabilities of edge servers alone are not sufficient.

## 8. Model Optimisation for Edge Computing

The core principle of edge computing is to enable end devices to process the information they gather and respond accordingly, with a minimal delay. However, some state-of-the-art applications utilise complex and computationally expensive DL models. Therefore, model optimisation is essential for deploying these architectures fully on edge devices, rather than offloading computation to an external server. Several metrics are essential for evaluating the performance, efficiency, and real-time capabilities of gait recognition models on edge devices. For instance, consider the following:

- Latency: this measures the time taken to process and recognise gait patterns, which are crucial for real-time applications.
- Energy consumption: this quantifies power usage, highlighting sustainability and device longevity, particularly for battery-powered systems.
- Model accuracy: this, including metrics like precision, recall, and F1-score (detailed in [140]), evaluates the effectiveness of gait recognition under constrained conditions.
- Model size: this refers to the storage footprint of the model.
- Throughput: this is the number of frames per second processed, also known as FPS.

Different techniques have been proposed to optimise deployment on edge devices. This section will cover the following optimisation methods: model design, pruning, quantisation, and knowledge distillation. For a more in-depth exploration of inference acceleration methods for edge computing, see [145,146].

### 8.1. Model Design or Selection

Edge AI researchers often prioritise designing DNN models with fewer parameters to minimise memory usage and execution delays while maintaining high accuracy. This is particularly important when designing DNN models for devices with limited computational capacity [145]. For example, Iandola et al. [147] proposed SqueezeNet to offer advantages like reduced communication across devices during training, less bandwidth consumption during model export, and the feasibility of deployment on embedded hardware, all while preserving the accuracy of AlexNet-level networks. The authors compressed SqueezeNet to less than 0.5 MB, achieving a 50-fold reduction in parameters compared to AlexNet. Similarly, Fang et al. [148] proposed Tinier-YOLO to minimise model size and improve both detection accuracy and real-time performance. Tinier-YOLO resulted in a model size of 8.9 MB, four times smaller than Tiny-YOLOv3. It achieved a mean average precision of 65.7% and 25 FPS real-time performance on a Jetson TX1.

### 8.2. Pruning

The pruning technique involves setting redundant or less important neuron parameters to zero without significantly impacting the accuracy of the results [149]. This strategy is widely used in DL model optimisation to reduce memory size and computational complexity. Recently, Yu et al. [150] proposed EasiEdge, a global pruning method to compress and accelerate DNNs for efficient edge computing. By adopting an alternating direction method of multipliers (ADMM) to decouple the pruning problem into a performance-improving problem and a global pruning sub-problem, their method achieved remarkable results. When pruning 80% of filters in VGG-16, the accuracy dropped by only 0.22%, and the GPU latency on a Jetson TX2 decreased to 0.19 ms. Furthermore, Woo et al. [151] presented “zero-keep filter pruning” for creating energy-efficient DNNs. This strategy replaces small values with zero and prunes the filter with the least number of zeros, thus maximising the number of zero elements in filters. Increasing the number of zero elements is expected to reduce the significant power/energy consumption associated with multiplication calculations through the use of a zero-skip multiplier.

### 8.3. Quantisation

Fundamentally, quantisation is the process of approximating continuous data, whether a single value or a series, using a set of integers. In the context of edge computing, parameter quantisation converts existing floating-point DNN parameters to lower-bit values. This eliminates expensive floating-point operations, thereby reducing the model’s computational weight and cost [145]. In this context, Zebin et al. [152] presented in 2019 a CNN model for classifying five activities of daily living. Their model used raw accelerometer and gyroscope data from a wearable sensor as input. Through model optimisation, they quantised weights per channel and activations per layer to 8-bit accuracy after training. This resulted in a CNN architecture with classification accuracy within 2% of floating-point networks. Notably, weight quantisation was responsible for nearly all of the size and runtime reductions in the improved model. Later, in 2021, Wardana et al. [135] used four distinct post-quantisation approaches provided by the TensorFlow Lite framework to develop an efficient model for edge devices: dynamic range quantisation, float16 quantisation, integer quantisation with float, and complete integer quantisation. Among these, full-integer quantisation yielded the lowest execution time, with latencies of 2.19 s and 4.73 s for Raspberry Pi 4B and Raspberry Pi 3B+, respectively.

### 8.4. Knowledge Distillation

Knowledge distillation is a technique in which a smaller, simpler model (the “student”) is trained to mimic the behaviour of a larger, more complex model (the “teacher”) [153]. The objective is for the student model to approximate the function learned via the teacher model, enabling powerful models to be deployed on resource-constrained devices where running the full-sized teacher model would be impractical. Moreover, smaller models are faster and require less memory, making them suitable for real-time applications. Common distillation strategies include logits distillation (the student learns to mimic the output probabilities of the teacher), feature distillation (the student learns to mimic intermediate layer activations of the teacher), and relationship distillation (the student learns to mimic the relationships between different neurons or layers inside the teacher). Recently, Li et al. [4] proposed applying knowledge distillation to CVGR systems, introducting multi-teacher joint knowledge distillation (MJKD) to address challenges in cross-view gait recognition. Specifically, MJKD uses multiple teacher models to train a lightweight student model, improving its ability to extract gait features and recognise individuals. Experiments on the CASIA-B dataset [23] demonstrated MJKD’s effectiveness, achieving 98.24% accuracy while reducing model complexity and computational cost.

## 9. Gait Recognition on the Edge

Gait recognition systems based on edge computing are a relatively new field, with few academic works addressing this combination (shown in Table 8). In contrast, multiple companies now offer gait recognition commercially, centred on a cloud-server architecture [154,155]. In the realm of CVGR systems, Tinini et al. [131] proposed a pioneering advance combining edge computing and model-free gait recognition, laying the groundwork for developing more robust and scalable real-time CVGR systems. Nevertheless, to consider a real-life application of this technology and achieve beneficial applications for society, much work remains to be done regarding the correct classification rate, different viewing angles, and robustness against intra-class and environment variations.

In 2022, Ruiz-Barroso et al. [156] conducted a comprehensive evaluation of hardware and software optimisations for deploying gait recognition models on embedded systems. They analysed the energy consumption and performance of three distinct models on two popular embedded platforms: the NVIDIA Jetson Nano 4 GB and the NVIDIA Jetson Xavier AGX. The study found that targeted hardware and software optimisations (specifically, pruning and quantisation techniques) significantly enhanced execution times, achieving a 4.2× speedup compared to the baseline models. Moreover, these optimisations resulted in a substantial improvement in energy consumption, with the deployed models consuming up to 5.4 times less energy.

**Table 8:** Summary of recent gait recognition systems for person identification approached with edge computing.

Year & Reference	Sensor	Gait Representation/Preprocessing	End Device	Gait Feature Classification Model	Metrics
2024 [157]	OAK-D Camera	GEI & Semantic Segmentation	Jetson Nano	CNN	29 FPS
2024 [13]	Accelerometer/Gyro	2D FFT	B. Akida/Arduino	CNN	0.97 accuracy
2023 [33]	FMCW-MIMO Radar	4D Point cloud videos	Laptop	Transformer	0.91 accuracy
2022 [156]	RGB Camera	GEI	Jetson Nano/AGX	2D/3D CNN	↓ 4.2 secs per inference
2022 [158]	Inertial sensor	Gait Feauture Map	Cellphone	CNN+LSTM	0.97 accuracy
2021 [131]	OAK-D Camera	MGEI & PCA	Jetson Nano	MobileNetv2, CNN	0.96 accuracy
2021 [11]	Floor sensors	Spatiotemporal signals	Raspberry Pi	CNN	0.91 f1-score

Recently, in 2023, Ma and Liu [33] introduced a new method for gait recognition using millimeter-wave radar. Specifically, they proposed a spatial–temporal network that processes 4D radar point cloud videos to capture the motion dynamics of walking people. The network employs PointNet to extract spatial features from each frame and then models the spatial–temporal information using a transformer layer. Experimental results demonstrate that the proposed network outperforms similar methods based on 4D radar reconstruction. Importantly, its ability to function under low-resource setups opens the door for real-time surveillance systems using 4D gait representations.

Deploying a CVGR system in real-world edge environments presents several significant challenges, which can be broadly categorised into three areas: variations in gait, environmental challenges, and security challenges. In the following sections, we will describe each of these challenge groups and discuss potential solutions proposed in the literature. Our goal is to simplify the decision-making process for those seeking to bring this biometric technology into real-world applications.

### 9.1. Variations in Gait

Changes in clothing, footwear, carrying objects, walking speed, and emotional state can alter a person’s gait pattern, making consistent recognition challenging. For example, a heavy coat or backpack can obscure the body’s silhouette and impact limb movement [1]. To address these challenges, researchers are exploring various strategies, such as the following:

- Developing robust feature extraction techniques and improving feature representations, such as utilising partitioned gait energy images [84] or adopting DL architectures [99], to reduce sensitivity to such variations.
- Employing view transformation models to normalise gait patterns across different perspectives [101].
- Creating synthetic gait data with controlled covariate gait variations [108,159].
- Implementing federated learning [160]—a distributed approach to data collection, model training, and weight sharing—for multi-view gait recognition.

Similarly, variations in viewpoint pose a common challenge for CVGR systems deployed on edge devices. Since these devices can be installed at different heights or with varying camera angles, even minor changes in setup can lead to perspective distortions that hinder the consistent comparison of gait features. Notable works addressing this issue include studies on gait recognition under view-invariant scenarios [113] and the use of GANs for covariate-aware gait synthesis [159].

### 9.2. Environmental Challenges

Environmental variations between laboratory and real-world settings can encompass a wide range of challenges that significantly impact the performance of CVGR systems. For instance, basic changes, such as walking surfaces (e.g., uneven terrain and stairs), can make it difficult to extract reliable gait features. Furthermore, different lighting and shadows are common issues for CVGR systems under real-world conditions. In such cases, applying a gamma filter, contrast stretching, or other image processing techniques can enhance the input image. Another frequent problem is the presence of occlusions (including people walking behind objects). To develop robust CVGR systems, gait recognition under occlusion scenarios has been developed for several years. For example, researchers in [161] recently proposed the combination of a VGG-16 model to detect occlusions and an LSTM model to reconstruct occluded frames in the sequence. In 2023, Xu et al. [6] introduced a technique for aligning silhouettes, which involves flexibly estimating the spatial extent of elements causing occlusion.

Moreover, real-world deployments often involve cameras with limited resolution, frame rate, or placement, leading to noisy and incomplete gait data. Addressing these real-world complexities requires developing robust algorithms and acquiring diverse training data that reflects such variability, as proposed in [108,118,159]. These advancements aim to improve the adaptability and robustness of gait recognition systems in real-world setups and under varying conditions.

### 9.3. Security Challenges

As emerging biometric technologies continue to gain widespread adoption, so, too, do the methods employed to breach their security. Automated biometrics, often utilised in access control and surveillance applications, are not immune to these challenges. Consequently, a more profound security analysis is imperative for the secure deployment of gait recognition systems at the edge. The following subsections explore three of the most frequent approaches to breaking CVGR systems and their potential countermeasures.

#### 9.3.1. Person Physical Impersonation

From the early 2010s, despite initial promising results, silhouette-based gait recognition systems raised concerns within the scientific community regarding their effectiveness as a biometric method. This prompted several investigations to test their vulnerabilities by having subjects mimic the walk style or clothing of others. For example, in 2012, Hadid et al. [162] published the first analysis of the spoofing susceptibility of such systems. They focused on experimenting with clothing and shape impersonation for two gait recognition systems, UOULU and USOU. These systems were originally designed to extract and process 2D and 3D visual data of walkers from a fixed viewpoint in a tunnel. The researchers then had 22 subjects walk through the same tunnel with similar clothes and body shapes, subsequently calculating the similarity between the recognised gaits. They concluded that, while silhouette-based recognition systems were indeed susceptible to spoofing, they were not as easily compromised as face or fingerprint recognition systems.

In 2015, Hadid et al. [163] proposed countermeasures to avoid physical spoofing. Their main countermeasure divides each silhouette within a frame sequence into multiple horizontal portions and computes local binary patterns from three orthogonal planes (LBP-TOP) for each portion. These portions are then combined into a single histogram vector, which is compared to determine whether the portions belong to the same person.

#### 9.3.2. Spoofing via Synthetic Data Generation

Recent advancements in DL-based synthetic data generation techniques, particularly autoencoders and GANs, have enabled the automated creation of fake human data such as face images, voice audio, and motion videos. In this context, Jia et al. [164] explored a spoofing method to compromise gait recognition systems. They proposed a GAN-based approach to render target videos on a given scene image from walking sequences. The focus was on generating realistic synthetic videos with good visual effects while also transmitting sufficient information to deceive gait recognition systems. Their experiments with GaitSet [92] and a CNN–gait-based approach validated the method’s effectiveness. Similarly, Hirose et al. [165] proposed a method to transform gait genuine clones (GGCs) from actual walking people into gait silhouette clones (GSCs) using a multiple autoencoder approach. To address potential security concerns, they also presented a supervised learning method to discriminate between genuine and synthetically generated gait silhouettes.

#### 9.3.3. Silhouette Poisoning for Misclasification

Similar to other DL-based biometric systems, gait recognition is also vulnerable to adversarial attacks. These attacks involve adding noise, in the form of patches or pixels, to a gait video, causing the DL models to misclassify the sample. For example, in a CVGR-based entrance security system compromised by such noise, the model might mistakenly recognise an unknown person as someone authorised to enter. To address this, Maqsood et al. [166] recently presented an approach to locate strategic sections in GEIs for adding imperceptible noise using gray wolf optimisation [167]. Their findings revealed that high-frequency textures are the most effective locations for such noise.

To enhance privacy in gait recognition, researchers in [168] proposed a privacy-preserving approach to anonymise human gait in videos by deforming silhouettes. They demonstrated that publicly available videos of people walking could be used to identify them without their knowledge and proposed deforming extracted gait silhouettes and replacing the original visual information in the videos with the modified ones. This resulted in a significant reduction in recognition accuracy from 100% to 1.57%, highlighting the approach’s potential to enhance privacy for individuals, albeit at the cost of model accuracy.

## 10. Discussion and Future Work

### 10.1. Trends and Challenges in Computer Vision-Based Gait Recognition Systems

Advances in CVGR systems are showing promising results, increasing the trustworthiness of this biometric technology. Moreover, the widespread use of closed-circuit television (CCTV) and low-cost cameras presents significant potential for integrating CVGR systems to enhance surveillance in public and private settings. However, the pursuit of higher precision has led to increasingly complex and resource-intensive models, often requiring deployment in high-performance computing environments. This limits the technology’s accessibility in scenarios with unstable internet connections or limited server capacity. Edge computing architectures offer a promising solution by distributing inference capabilities, thus addressing this limitation. As discussed in Section 8, several studies have demonstrated the effectiveness of edge computing architectures, highlighting opportunities to apply these to CVGR systems.

In terms of models, distance-based gait sequence classifiers remain the most straightforward and resource-efficient approach. However, machine learning becomes crucial when generalisation is critical for a CVGR project. Figure 1a,b show a trend towards employing DL models in CVGR systems, with CNNs, RNN, LSTM, GAN, CapsNets, and transformers being the most frequent. Despite remarkable advancements in DL-based CVGR systems, their real-world application in uncontrolled environments remains challenging, although recent works and datasets, such as [14,16], demonstrate promising results. For example, recent results obtained by DL models with challenging gait sequence variations, such as GPGait [7] and SkeletonGait [43], highlight opportunities for model-based approaches. On top of that, novel gait recognition technologies such as depth sensing, stereo vision, 3D laser scene reconstruction, pose estimation, optical flow, and graph-based models hold promising research threads for future investigations.

Newer technologies have yet to overcome challenges such as variations in illumination. Changes in lighting due to time of day, weather, and shadows can distort silhouette- or appearance-based features, affecting gait extraction. Another challenge is occlusions, where objects, other people, or the environment obscure body parts, leading to incomplete gait data. Viewpoint variation is also critical. Unlike controlled indoor setups with consistent camera angles, outdoor environments often involve dynamic viewpoints, making it difficult to match gait features across different perspectives when implementing CVGR systems [7].

### 10.2. Edge Computing for CVGR Systems

Given the computational complexity of gait data acquisition, representation, dimensionality reduction, and recognition, deploying a DL-based workflow on a single device can be demanding. However, a distributed edge computing approach offers a solution by enabling task distribution across multiple devices. As detailed in Section 7, several architectures for distributing inference are currently used in academia and industry. These architectures are applicable to CVGR systems and can be further researched for optimisation when implementing CVGR systems coupled with new sensing modalities (3D laser and stereo vision) or feature representations (skeleton maps, point clouds, and graph-based models), which still require high-capacity servers. Nevertheless, the data- and resource-intensive nature of DL necessitates larger datasets and more complex pipelines, making it challenging to embed these pipelines in resource-constrained environments.

This article revealed several promising technologies for developing CVGR systems suitable for edge devices. For example, 2D imaging currently offers advantages over 3D scene reconstruction technologies (depth sensing, stereo vision, and 3D lasers). Most model-free gait feature representations discussed in Section 4.2 are advantageous for deployment in edge computing architectures, while the growing precision of model-based approaches holds promising opportunities for future investigations. Furthermore, the nature of graph-based models shows promise for embedding in low-resource devices; however, they currently require further optimisation methods (detailed in Section 8). Finally, dimensionality reduction techniques remain relevant due to the high dimensionality of gait data, especially when using multi-modal and multi-camera approaches to gait recognition.

Additionally, the increasing availability of powerful edge AI development kits (detailed in Table 6), such as the NVIDIA cards, Google Coral Dev Board, Qualcomm Snapdragon AI Development Kit, and Khadas VIM, is opening opportunities to embed more complex pipelines at the edge. These development cards can be supported by USB AI modules designed to execute individual computer vision models. These modules, including the OAK-D, Hailo-8, and Coral USB Accelerator (among others detailed in Table 7), can perform tasks such as person detection, silhouette semantic segmentation, or body keypoint extraction to optimise the CVGR pipeline. Many of these devices enable parallel computing through dedicated chips (GPUs, VPUs, and TPUs) and are suitable for model compression and optimisation using tools such as NVIDIA TensorRT, Intel OpenVINO, and TensorFlow Lite. Table 8 showcases examples of CVGR systems designed for resource-constrained devices, highlighting a growing research trend in combining CVGR and edge computing. This trend is further supported by the multiple successful cases of computer vision systems combined with edge computing, as shown in Table 5.

### 10.3. Applications of CVGR Systems on the Edge

Beyond security applications, edge-based CVGR systems hold significant potential for healthcare, sports, and human–computer interaction. In healthcare, this approach could improve rehabilitation monitoring for neurological disorders (e.g., Parkinson’s disease), moving beyond traditional offline analysis to provide real-time feedback. For instance, authors in [34,169] implemented offline CVGR systems to assess patients and identify abnormalities that may indicate health risks or a mental health decline. Yet, by analysing subtle gait changes in real time, edge devices can further enhance these assessments, supporting personalised rehabilitation plans and enabling continuous monitoring for at-risk individuals without relying on expensive centralised infrastructure. Another promising application of edge-based CVGR is in fall prediction [170]. While most CVGR-based fall detection systems have primarily focused on offline analysis, on-device computing can enable continuous surveillance for fall prevention of people who require special care.

In sports, a CVGR system can easily gather and process precise movement data, offering insights about an athlete’s biomechanics (e.g., stride length, stride frequency, and gait phase) to refine athletic technique and detect movement disorders early on. Moreover, CVGR systems deployed on the edge can offer unique advantages for gait anomaly identification: continuous analysis, long-distance capture, simultaneous monitoring of multiple athletes, non-invasiveness, and resistance to imitation. A related approach was researched by Freire et al. [171], who applied machine learning to analyse gait patterns inside a gaming robot, providing real-time feedback for improving athletic performance and assisting individuals with mobility challenges. Their findings, supported by a CVGR system deployed on the edge, can ease the creation of adaptive and precise sports training systems, optimising biomechanics continuously, and enabling effective rehabilitation processes.

Finally, applications for human–computer interaction include personalised interfaces and emotion recognition. For the first group, CVGR systems can recognise individuals to personalise the environment, enhancing user experiences in home automation. Related research by Chi et al. [172] explored user-centred robotics by deploying gait recognition into service robots for human-following tasks in dynamic, challenging environments. For emotion recognition, studies integrating 2D CNNs with pose estimation enabled real-time gait-based emotion inference, fostering advancements in assistive technologies [173].

## 11. Conclusions

Gait recognition is achieving increasingly robust results every year, thanks to advances in deep learning architectures. However, most recent approaches still rely on sending video of gait sequences to a central server for processing, which necessitates an internet connection and can be costly. In contrast, edge computing proposes collecting and processing data closer to the source on local devices, offering faster response times and enabling real-time monitoring. This trend, coupled with the availability of powerful mini-computers, makes it possible to run advanced CVGR systems directly on edge devices where the gait data are captured.

This paper has reviewed different edge computing architectures for CVGR, discussed their design and deployment, and explored the challenges and future possibilities of integrating these technologies. The paper emphasises methods suitable for low-resource devices and identifies areas where further research is needed. Significantly, the review highlights a research gap: the limited exploration of combining CVGR and edge computing. This integration has the potential to substantially improve gait recognition implementations, making them more accurate and suitable for real-world applications, including the continuous identification of individuals and patient health monitoring.

Future CVGR systems should explore modular frameworks that leverage edge computing. Distributing gait recognition tasks across multiple edge devices can significantly improve inference speed while maintaining accuracy and enhancing system robustness by capturing gait data from diverse viewpoints. This approach also provides a foundation for improved multi-modal or multi-view frameworks, where different end devices or sensors capture data of the same subject for subsequent recognition, offering immense potential for advancing gait recognition with improved accuracy and scalability.

## Figures and Tables

**Table 1:** Datasets for 2D Imaging-based gait recognition sorted by year.

Name & Reference	Year	Subjects	Sequences	Views	Variations	Environment
CASIA-E [14]	2022	1014	778,752	26	Dressing, Carrying, Walking Style, Gender, Age	Outdoor
ReSGait [15]	2021	172	870	1	Clothing, Carrying, Trajectories, Gender	Indoor
GREW [16]	2021	26,345	128,671	882	Clothing, Carrying, Occlusion, Viewpoint, Background	Outdoor
VersatileGait [17]	2021	11,000	1,320,000	44	Age, Gender, Walking Style	Unity3D
OU-MVLP * [18]	2020	10,307	268,086	14	Viewpoint	Indoor
KY4D * [19]	2014	42	84	16	Curve	Indoor
SOTON * [20]	2011	300	5000	12	Viewpoint	Indoor
SAIVT-DGD [21]	2011	35	700	1	Speed, Carrying, Shoes	Indoor
CASIA-C [22]	2006	153	1530	1	Speed, Walking Surface	Outdoor
CASIA-B [23]	2006	124	13,640	11	Clothing, Carrying, Walking Surface	Indoor
CASIA-A [24]	2003	20	240	1	Walking Direction	Outdoor
UCSD [25]	1999	6	42	1	Clothing, Carrying	Outdoor

* The dataset comes in different versions. The specified details pertain to its most recent version.

**Table 2:** Key differences between handcrafted and deep learning (DL) gait feature representations.

Aspect	Handcrafted Features	Deep Learning Features
Feature Design	Manually designed using domain knowledge.	Automatically learned from data.
Computational Complexity	Generally lower complexity, faster to compute.	Higher computational complexity, requires more resources.
Generalisation Ability	Limited generalisation, often tailored to specific conditions.	Better generalisation, more robust to diverse conditions.
Adaptability	Requires redesign for new tasks or environments.	Learns features adaptively from data.
Data Requirement	Can work with smaller datasets.	Requires large amounts of labelled data for training.
Interpretability	Easier to interpret due to human design.	Harder to interpret, features are learned in a black-box fashion.

**Table 3:** Model-based gait feature representations and their frequency of use in recent research. Identifying the earliest papers introducing each approach can be challenging.

Gait Feature Representation	Year	Pros	Cons	Frequency of Use	Recent Applications
Graph-Based Models	2021	Effective for capturing relationships between joints, capable of encoding spatial and temporal dependencies.	Computationally intensive, requires large datasets, can be sensitive to noise in joint detection.	Emerging, increasingly popular	[40,41,42]
Pose Estimation	2016 (DL-based)	Captures human joint movements with high granularity, robust to appearance changes, clothing, and background noise.	Sensitive to inaccuracies in joint detection, requires high-quality input, limited in occlusion cases.	Increasingly frequent	[7,31,43]
Skeleton Maps	2010s	Simple representation of body joints, efficient for machine learning models, invariant to appearance changes.	Can miss subtle gait dynamics, reliant on accurate joint detection, struggles with occlusions or missing joint data.	Moderately frequent	[16,43]

**Table 5:** Frequency of use of DL models for CVGR and their suitability for edge computing.

Model	Frequency of Use in CVGR Systems	Suitability for Edge Computing	Recent Applications for Edge Computing
CNN	High	Moderate, requires optimisation.	[130,131]
Autoencoder	Moderate	High, compact representations.	[132]
GAN	Moderate	Low, resource-intensive.	[133]
CapsNet	Low	Low, high complexity.	[134]
RNN	High	Moderate, LSTM/GRU optimisations required.	[135]
GNN	Moderate	Low, requires significant optimisation.	[119]
GCN	Moderate	Low, optimisation needed for real-time deployment.	[136]
Transformer	Increasing	Low, requires optimisation for edge devices.	[129]

**Table 6:** Computational capabilities of edge computing development cards, sorted by year of release.

Device	Release Year	GPU	CPU	RAM (Gigabytes)	Storage (Gigabytes)	Performance (TFLOPS)
Qualcomm Snapdragon AI Dev Kit	2024	Adreno 740	Qualcomm 8-core Kryo	16	512 GB NVMe SSD	4.6
Jetson Orin Nano	2023	1024-core Ampere + 32 Tensor Cores	6-core ARM Cortex-A78AE v8.2	8	16 eMMC/ Expandable	10
Khadas VIM4	2022	ARM Mali-G52 MP8	Hexa-core Amlogic A311D2 SoC	8	16 eMMC/ Expandable	3.2
Jetson Orin AGX	2022	2048-core Ampere + 64 Tensor Cores	12-core ARM Cortex-A78AE	32	64 eMMC/ Expandable	40
Jetson Orin NX	2022	1024-core Ampere + 32 Tensor Cores	6-core ARM Cortex-A78AE	16	128 eMMC/ Expandable	20
Xilinx Kria KV260	2021	No GPU FPGA Integrated	Quad-core ARM Cortex-A53	4	16 eMMC	1.4
Jetson Xavier NX	2020	384-core Volta + 48 Tensor Cores	6-core ARM Carmel	8	16 eMMC/ Expandable	1.3
Jetson Nano	2019	128-core Maxwell	Quad-core ARM Cortex-A57	4	16 eMMC/ Expandable	0.5
Khadas VIM3	2019	3-core ARM Mali-G52 MP2	Hexa-core Amlogic A311D	4	16 eMMC/ Expandable	0.8
Google Coral Dev Board	2019	Integrated GC7000 Lite Graphics	Quad-core ARM Cortex-A53	4	16 eMMC/ Expandable	0.25

All cards come in different versions, and the specified details are from their most capable and recent versions. For information on other versions, please refer to the official product website.

**Table 7:** Recent prebuilt devices for AI acceleration, sorted by year of release. Most of them can be connected via USB to edge computing development cards.

Device Name	Release Year	Type	AI Accelerator Type	Performance (TOPS)	Power (Watts)
Gyrfalcon	2022	USB Module	MPE	16	2
Intel Edge AI Box	2021	Prebuilt Box	Intel Movidius Myriad X VPU	4	20
Hailo-8	2021	USB Module	Hailo-8 Neural Processor	26	2.5
OAK-D	2020	Camera	VPU	1.4	5
Coral USB Accelerator	2019	USB Module	Edge TPU	4	2

## Abbreviations

The following abbreviations are used in this manuscript:

ANNs	Artificial neural networks
CNN	Convolutional neural network
CVGR	Computer vision-based gait recognition
DCT	Discrete cosine transform
DL	Deep learning
DNN	Deep neural network
DR	Dimensionality reduction
DWT	Discrete wavelet decomposition
ff-GEI	frame-by-frame gait energy image
GAN	Generative adversarial networks
GEIs	Gait energy images
GEnIs	Gait entropy images
GFI	Gait flow image
GHI	Gait history image
GNNs	Graph neural networks
GPU	Graphics processing unit
IoT	Internet of things
LDA	Linear discriminant analysis
LiDAR	Light detection and ranging
LSTM	Long short-term memory
MEI	Motion-energy image
MHI	Motion-history image
MSI	Motion silhouettes image
PCA	Principal component analysis
ML	Machine learning
RNN	Recurrent neural networks
t-SNE	t-distributed stochastic neighbor embedding
TFLOPS	Tera floating-point operations per second
TOPS	Tera operations per second
TPU	Tensor processing unit
VPU	Vision processing unit
